# Long-term outcomes of percutaneous closure of ventricular septal defects in children using different devices: A single centre experience from Egypt

**DOI:** 10.1186/s12887-023-04194-9

**Published:** 2023-07-31

**Authors:** Hala Elmarsafawy, Mona Hafez, Gehan A. Alsawah, Asmaa Bakr, Shaimaa Rakha

**Affiliations:** 1 Pediatric Cardiology Unit, Pediatrics Department, Mansoura University Faculty of Medicine, El Gomhouria St, Mansoura, Dakahlia Governorate 35516 Egypt; 2 Faculty of Medicine, New Mansoura University, New Mansoura City, Egypt

**Keywords:** Devices, Long-term, Percutaneous, Ventricular septal defects

## Abstract

**Background:**

The feasibility of percutaneous closure ventricular septal defects (VSD) in children has been previously proven. However, data on long-term outcomes are limited. We aim to evaluate the long-term outcome of our experience with percutaneous closure of VSD using various occluders.

**Methods:**

Retrospective institutional analysis of children who underwent transcatheter closure of perimembranous and muscular VSDs between September 2012 and February 2020. Patient demographics, procedural, and long-term follow-up data were comprehensively analyzed. Patients who lost to follow-up within two years post-procedure were excluded.

**Results:**

We identified 75 patients (54.7% males) with a median of 66 months (IQR, 46–96). The closure success rate at one year was 95.7%. Complete heart block was detected in two patients early post-procedure and resolved with steroids. The VSDs were perimembranous (52%), muscular (33.33%), and residual (14.67%). Implanted devices were Pfm Nit-Occlud LeˆVSD Coil (42.7%), Hyperion^TM^ VSD Muscular Occluder (28%), Amplatzer VSD muscular occluder (10.7%), Amplatzer Duct Occluder (14.7%), Occlutech Muscular VSD Occluder (2.7%), and Amplatzer Duct Occluder II (1.3%). No new arrhythmia or valve regurgitation was detected after two years post-procedure. Persisted complications on long-term follow-up included: residual shunting in 3(4%), mild tricuspid regurgitation in 2(2.7%), and aortic regurgitation in 2(2.7%), with one immediate post-catheterization mild aortic regurgitation worsened during follow-up, requiring surgical repair of VSD three years after device implantation. No deaths were reported.

**Conclusion:**

Long-term outcomes of pediatric transcatheter VSD closure using different devices are satisfactory. Post-procedural adverse events are limited, but long-term surveillance is necessary to monitor their progression.

## Introduction

Ventricular septal defect (VSD) is the most common congenital heart disease, accounting for 30% of all congenital cardiac malformations [1]. Closing VSD is sometimes needed because spontaneous closure may not occur with subsequent potential complications. Surgical closure remains the mainstay of treatment, especially for large defects. However, some defects are better approachable percutaneously [2].

The first VSD transcatheter closure was reported by Lock et al. in 1988, using a Rashkind double-umbrella device [3]. Since then, we witnessed huge advances in device technology and imaging modalities, and device closure has become widely adopted to close hemodynamically significant native or residual defects, thereby avoiding cardiopulmonary bypass and lengthy hospital stays [4–9].

The immediate and midterm results of percutaneous closure of VSD have been extensively reported, with well-established procedure safety and efficacy [10–16]. However, data on longer-term outcomes using variable occluders is still limited. Therefore, we aim to present our institutional experience with transcatheter VSD closure and evaluate the long-term outcomes of the procedure.

## Materials and methods

We performed a retrospective clinical data review of pediatric patients (≤ 18 years old) who underwent device closure of perimembranous and muscular VSD at our institution. The Institutional review board (IRB) of Mansoura University, Faculty of Medicine, Egypt, approved the study. Informed consent was obtained from the patient’s legal guardians. Patients who lost to follow-up within two years post-procedural were excluded from this study.

Indications for VSD closure at our institution were hemodynamically significant left-to-right ventricular shunt or a history of infective endocarditis related to the VSD. The hemodynamically significant VSD was defined by the presence of at least 3 of the following criteria: (1) overt heart failure, not improving with appropriate medication, (2) failure to thrive, predominantly due to the hemodynamic effects of the VSD, (3) recurrent respiratory infections, (4) Increased cardiothoracic ratio on chest X-ray, (5) left ventricular (LV) end-diastolic z-score on echocardiogram, indexed to body surface area of ≥ 2.0, and (5) estimated pulmonary to systemic blood flow ratio of > 1.5 at cardiac catheterization [6, 17].

Cases not eligible for closure were: (1) large defects ≥ 10 mm, (2) bidirectional or predominantly right to left shunt on color Doppler, (3) aortic valve prolapse or aortic regurgitation more than trivial, and (4) doubly committed VSDs or complex cardiac anatomy requiring surgical intervention.

### Procedure

All procedures were performed under general anesthesia, and fluoroscopy with transesophageal or transthoracic echocardiographic guidance. Intravenous antibiotic prophylaxis (cefotaxime or cefuroxime 50 mg/kg, up to 1 g) was administered before the procedure. Short venous and arterial femoral access was obtained, preferably on the right side. Intravenous Heparin (100 IU/kg, up to 5000 IU) was given to keep activated clotting time above 200s. First, routine right and left heart catheterizations were done to evaluate the pulmonary-to-systemic flow ratio (Qp/Qs). Then, left ventriculography and aortography, in lateral and 30° right anterior oblique/15° caudal projections, were performed to delineate the VSD anatomy and evaluate the presence of aortic valve prolapse or regurgitation.

A 4 or 5 French partly cutoff pigtail or Judkins right catheter was advanced to the left ventricle or ventricular outflow tract. Then, a retrograde or antegrade approach was used for VSD closure [18].

Available devices during the study period were: Amplatzer Duct Occluder I (ADO I), (Abbott, USA); Amplatzer Duct Occluder II (ADO II), (Abbott, USA); Amplatzer Muscular VSD Occluder (AMO), (Abbott, USA); Nit-Occlud Leˆ VSD coil (PFM Medical, Germany); Hyperion™ VSD Muscular Occluder (Comed B.V., Netherlands/Lepu MT Company, China); and Occlutech Muscular VSD Occluder (Occlutech GmbH, Germany).

Device selection depends on the defect morphology, location, relation to the aortic valve, the diameter of the defect, and device availability in the armamentarium. For native perimembranous and residual VSDs, ADO I was used in the presence of significant-sized aneurysms and a subaortic rim ≥ 5 mm. The ADO I right disc diameter was selected to be 2 mm more than the smallest VSD diameter. ADO II was used in defects with no aneurysmal tissue, diameter < 5 mm, and subaortic rim > 3 mm. The chosen ADO II waist diameter was 1 mm more than the smallest VSD diameter. Nit-Occlud Leˆ VSD coil was used in aneurysmal VSD with a diameter < 8 mm and subaortic rim ≥ 3 mm. The selected coil had a distal end at least twice the minimal VSD diameter on the right ventricular side and equal to or 1–2 mm greater than the left ventricular opening of VSD. Hyperion device was used in some cases with a defect to aortic valve distance >4 mm as the length of the device’s left retention skirt is 4 mm. For muscular VSDs, one of the muscular occluders was used (AMO, Hyperion, Occlutech), according to the device availability of an appropriate size in the catheterization lab. However, AMO was available throughout the duration when our patients had their VSD closure, but Hyperion use in our patients was started in 2018 and Occlutech in early 2020. The size of the muscular occluder depends on the device’s waist diameter, which was chosen to be 1–2 mm larger than the VSD.

Through the long sheath, the device was deployed under fluoroscopic guidance. Before release, left ventriculography was performed 10 min after device implantation to verify the occluder position, residual shunt, and valvular condition. After the procedure, oral aspirin 3–5 mg/kg, up to 100 mg daily, was prescribed for six months in all patients.

Successful closure was defined as the absence or trivial residual shunting. Trivial shunting was defined as a minimal signal in color Doppler without a complete signal on pulsed/continuous Doppler [19].

### Post-procedural follow-up

Follow-up evaluation was performed on day one post-procedure, three and six months, then every 12 months. Device position, VSD residual shunt, and valvular condition were evaluated by transthoracic echocardiography. Holter monitoring was performed when an abnormality was suspected on 12-lead electrocardiography (ECG).

### Last follow-up visit

The time elapsed from the intervention was documented. Two-dimensional, color flow, Doppler, and three-dimensional echocardiography were performed to document residual flow, device position, aortic valve morphology/function, and adverse events. The presence of heart block or abnormal rhythm in the last follow-up was looked at.

### Complications

Life-threatening adverse events or those requiring surgical management were defined as serious complications. Adverse events requiring medical or transcatheter intervention were defined as major complications. Complications that did not require specific management were termed minor complications [19].

### Statistical analysis

Statistical analyses were performed using SPSS, version 25 (IBM, Armonk, NY, USA). Categorical variables were reported as frequency and percentage, while continuous variables were represented as median with interquartile range (IQR). Statistical analyses for continuous variables were conducted using Kruskal-Wallis test, while Chi-square test and Fisher’s exact test were used for categorical variables as appropriate. A p-value < 0.05 was considered statistically significant. All reported P values are two-sided.

## Results

**Fig. 1:**
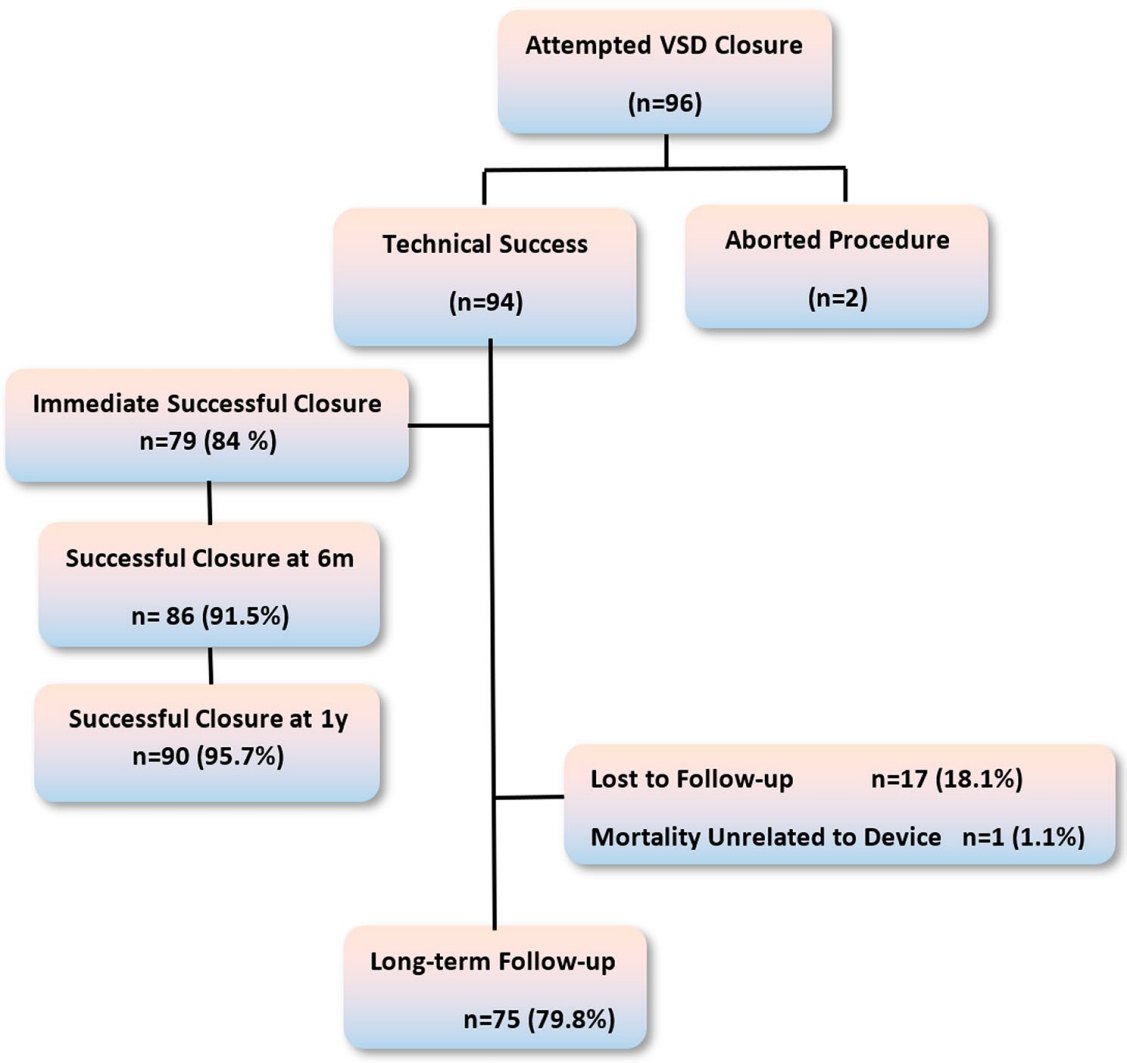
Chart demonstrating the study’s cohort of patients. **n**: Number of patients, **m**: months, **y**: year

From September 2012 to February 2020, 96 patients had an attempted closure of VSD. Implantation was initially achieved in 94/96 patients with a technical success of 97.9%. Of the 94 cases, most cases were treated successfully with a single procedure; nevertheless, four patients required a second device insertion within the same procedure; one due to immediate embolization after release, another due to partial device herniation before release, and the other two because of significant residual shunt verified by ventriculography before device release. After the device release, the closure immediate success rate was 79/94 (84%), which increased at six months to 91. 5%, and reached 90/94 (95.7%) at one year of follow-up, with no further increase on subsequent follow-up. Long-term follow-up was possible in 75/94 (79.8%) patients with inserted devices (see Fig. 1). Eighteen cases were lost to follow-up (none had immediate complication), with one death unrelated to the procedure (traffic accident).

**Fig. 2:**
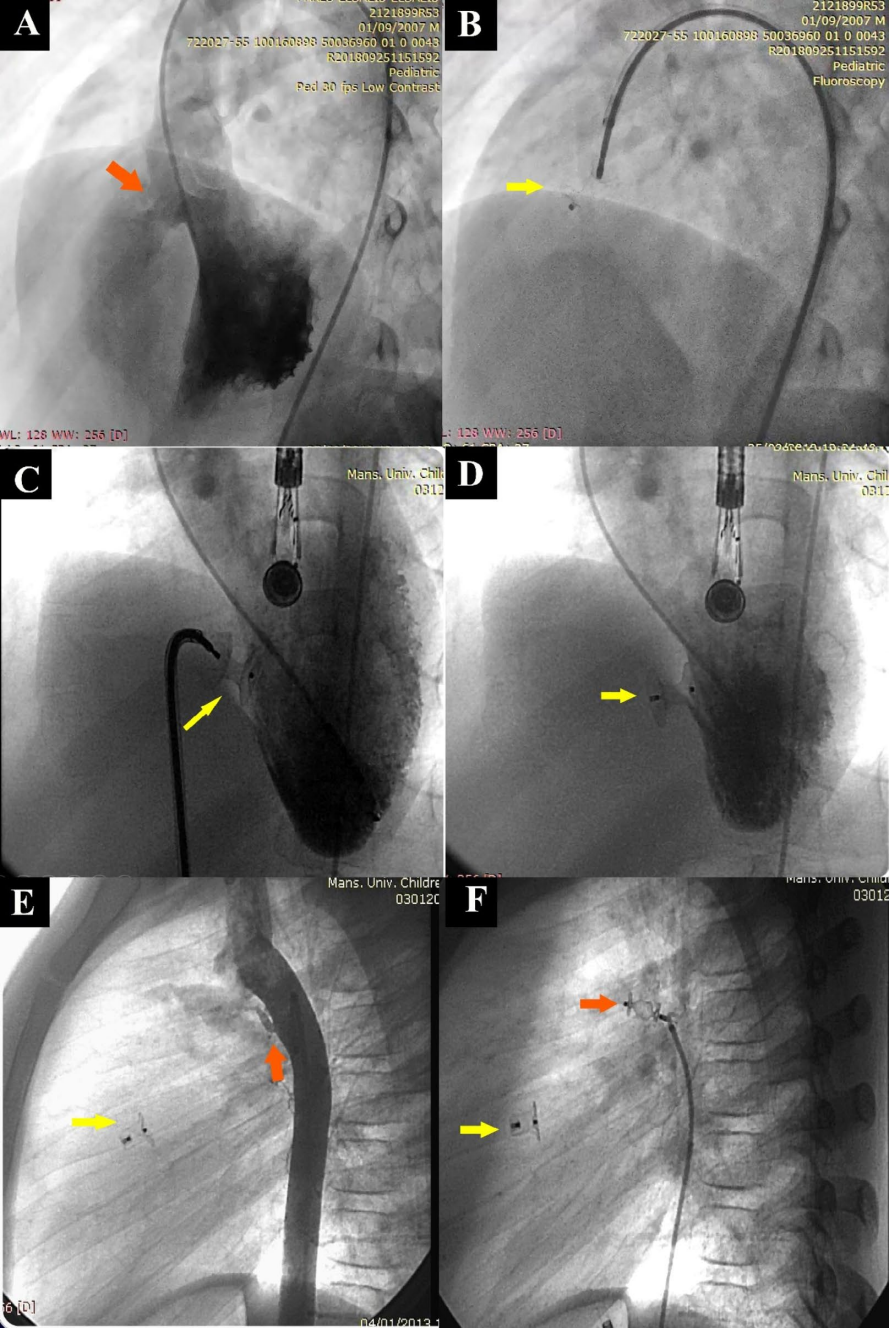
**(A)** LV angiography (60° left anterior oblique with 20° cranial) to determine the location and size of VSD (red arrow). **(B)** Retrograde approach for implanting Hyperion device across VSD (yellow arrow). **(C)** Antegrade approach for implanting Amplatzer muscular occluder device across VSD (yellow arrows) with LV angiography before the occluder released, LV angiography showed no residual shunt and satisfying position and shape of the occluder. **(D)** LV angiography after Amplatzer muscular occluder release with no residual shunt. **(E)** Aortic angiography demonstrating small elongated PDA (red arrow) after closing VSD with ADO I (yellow arrow). **(F)** Two devices are seen in place; Vascular plug II closing PDA (red arrow) and ADO I across VSD (yellow arrow)

Table 1 demonstrates the demographic and cardiac lesion characteristics of the included patients. The median follow-up duration (IQR) was 66 (46–96) months with a minimum follow-up of 28 months, and the highest follow-up duration was 115 months (9.6 years). The study included 75 patients; 54.7% were males. The median (IQR) age of cases at intervention was 9 (7–11) years (ranging from 3 to 17 years), and the median weight (IQR) was 30 (21–43) kg (ranging from 12 to 77 kg). Most patients had native perimembranous VSD in 39 (52%), followed by muscular VSD in 25 (33.3%), and residual VSD post-cardiac surgeries in 11 (14.7%). Of the residual VSD cases, 6 had right bundle branch block (RBBB) pre-catheterization. A concomitant cardiac lesion was present in 19/75 (25.3%); one patient had a PDA closure transcatheter in the same sitting (see Fig. 2), and another had pulmonary valvuloplasty simultaneously with VSD closure.

**Table 1:** Demographics and cardiac lesion characteristics of the included patients in the long-term follow-up

Parameter		Patients* n (%) or median (IQR)
**Follow-up duration (Months)**		66 (46–96)
**Sex**	Male	41(54.7)
**Age at intervention** **(years)**		9 (7–11)
	1-5y	10 (13.3)
	6-10y	38 (50.7)
	11-18y	27 (36)
**Weight at intervention (Kg)**		30 (21–43)
	10-20 kg	17 (22.7)
	21-30 kg	22 (29.3)
	31-40 kg	14 (18.7)
	41-50 kg	15 (20)
	> 50 kg	7 (9.3)
**VSD Types**	***Native Perimembranous***	39 (52)
	***Native Muscular***	25 (33.3)
	***Residual postsurgical*** • s/p peri-membranous VSD • s/pTOF • s/p DORV, subaortic VSD • s/p AVSD	11(14.7) 5 4 1 1
**Concomitant cardiac lesion**		19 (25.3)
	***Small ASD/PFO***	13
	***Mild to moderate PS***	4
	***Small PDA***	2

**ASD**: atrial septal defect, **AVSD**: atrioventricular septal defect, **PFO**: patent foramen ovale, **PS**: pulmonary stenosis, **TOF**: tetralogy of Fallot, **VSD**: ventricular septal defect

*Data are expressed as median (interquartile range) or number (Percentage)

Procedural and device data of the included patients in the study are demonstrated in Table 2. Most transcatheter VSD occluders were implanted through an antegrade approach in 53.3%, while the retrograde approach was used in 46.7% (see Fig. 2). Moreover, the most used echocardiographic guidance was transesophageal in 85.3% of cases. Pfm Nit-Occlud Leˆ VSD Coil was the most used device in 32 (42.7%) cases, especially for perimembranous VSD 26 (66.7%), followed by Hyperion^TM^ VSD Muscular Occluder 21(28%), particularly for the muscular VSD type.

**Table 2:** Procedural and device data of the included patients in the study

Parameter			Value* n (%) or median (IQR)
**Angiographic Defect diameter (mm)**			6.4 (4.5–7.8)
**Procedural time (in minutes)**			118 (90–125)
**Fluoroscopy time (in minutes)**			23.5 (19-45.5)
**Pulmonary artery mean pressure (mmHg)**			20.5 (17.1–23.4)
**Approach**	• **Antegrade**		40 (53.33)
	• **Retrograde**		35 (46.67)
**Echocardiographic** **guidance**:	• **TEE**		64 (85.3)
	• **TTE**		11 (14.7)
**Device size**			8 (6–10)
**Types of Used Occluders**	• **Pfm Nit-Occlud Leˆ VSD Coil**		32 (42.7)
	• **Hyperion**^**™**^**VSD Muscular Occluder**		21 (28)
	• **Amplatzer Muscular Occluder (AMO)**		8 (10.7)
	• **Amplatzer Duct Occluder (ADO I)**		11 (14.7)
	• **Amplatzer Duct Occluder (ADO II)**		1 (1.3)
	• **Occlutech Muscular VSD Occluder**		2 (2.7)
**Occluders used for Each VSD type**	**Perimembranous** 39 (52)	Coil	26 (66.7)
		Hyperion	6 (15.4)
		ADO I	6 (15.4)
		ADO II	1 (2.6)
	**Muscular** 25(33.3)	AMO	8 (32)
		Hyperion	15 (60)
		Occlutech	2 (8)
	**Residual** 11(14.7)	Coils	6 (54.5)
		ADO I	5 (45.5)

**ADO**: Amplatzer duct occluder, **AMO**: Amplatzer Muscular Occluder, **TEE**: transesophageal echocardiography; **TTE**: transthoracic echocardiography; VSD; ventricular septal defect

*Data are expressed as mean ± SD or median (Interquartile range) or number (Percentage)

**Fig. 3:**
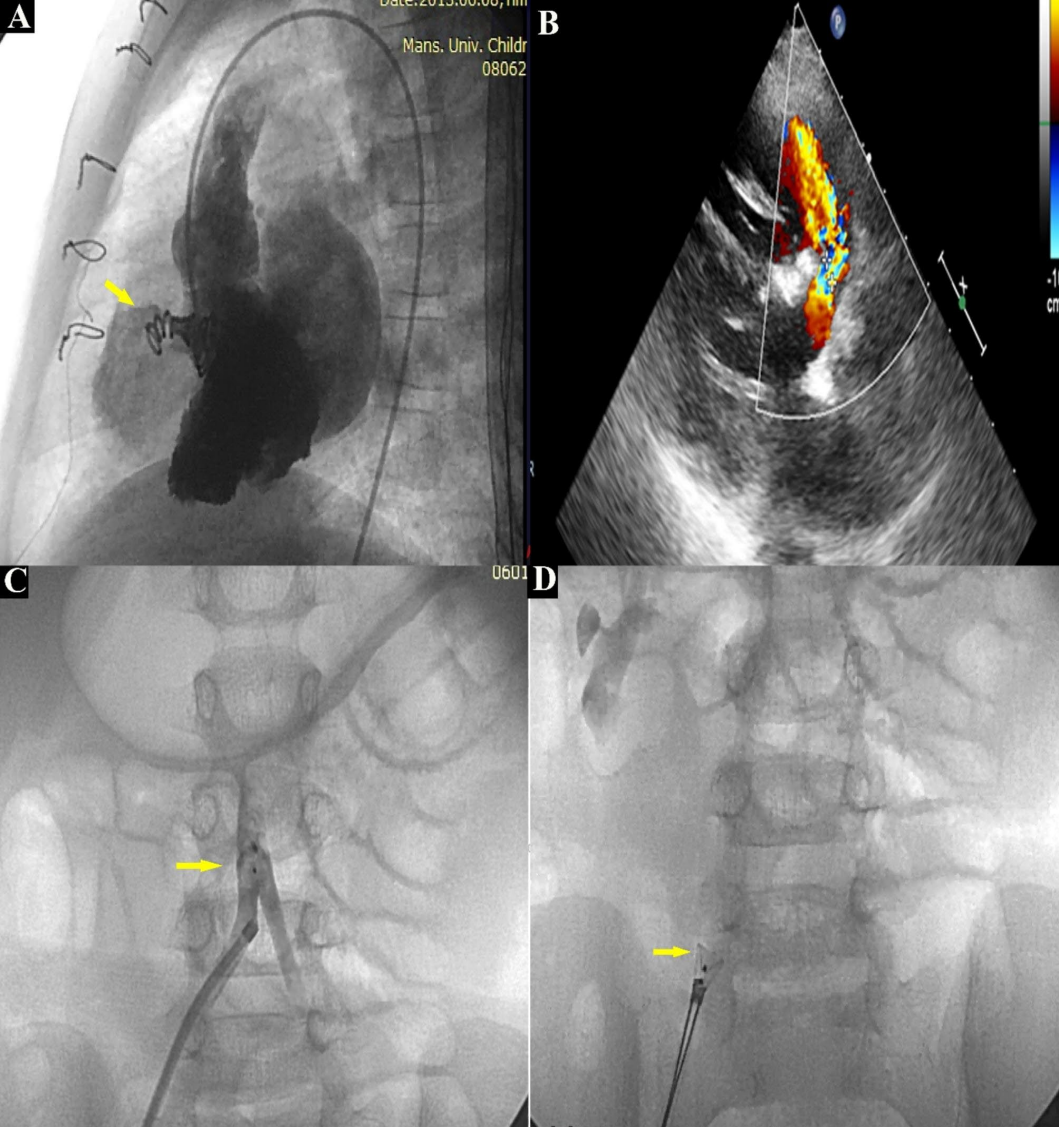
Early complications of VSD device closure **(A)** LV angiography demonstrating coil in place across subaortic perimembranous VSD with mild residual shunt **(B)** Post-procedure transthoracic echocardiography showing residual shunt **(C)** Hand injection in right common iliac artery demonstrating embolized ADO I into the bifurcation of the aorta without causing obstruction **(D)** ADO I embolized Device was successfully snared. Yellow Arrow is pointing at the device

Details of early post-procedural and long-term mortality and morbidity are documented in Table 3. Early Minor complications were detected in 28.7% of cases. One of the major complications encountered was device embolization. It occurred in two patients; the first had a Hyperion device embolized immediately upon release, but it was snared and replaced with a larger device. The other was an ADO I device embolized one day after the procedure, which was retrieved transcatheter with surgical closure later based on the parents’ request (see Fig. 3). Access-related limb ischemia requiring systemic heparin infusion occurred in one child.

**Table 3:** Short- / long-term procedure-and device-related morbidity and mortality

Parameter			Value
Short-term postprocedural Complications (n = 94)	• ***Minor*** 27/94(28.7)	• Access-related Hematoma	2 (2.13)
		• Access-related Bleeding	1 (1.1)
		• Residual shunt (trivial/ mild)	14 (14.9)
		• Infrequent arrhythmia	2 (2.1)
		• New Tricuspid regurgitation (mild)	5 (5.3)
		• New aortic regurgitation (trivial/ mild)	3 (3.2)
	• ***Major*** 5/94 (5.3)	• Device Embolization	2 (2.1)
		• Access-related limb ischemia	1 (1.1)
		• Transient Complete CHB	2 (2.1)
		• Hemolysis	-
	• ***Serious***		-
Short-term Mortality			-
Long-term Complications: (n = 75)	• ***Minor*** ***5/75 (6.67)***	• Residual flow on the last follow-up (mild) • Mild Tricuspid regurgitation • Trivial Aortic regurgitation	3 (4) 2 (2.7) 1 (1.3)
	• ***Serious*** ***1/75 (1.3)***	• Severe Aortic regurgitation • Infective endocarditis/ Erosion	1 (1.3) -
Long-term Mortality			-

* Data are expressed as number (Percentage)

Complete atrioventricular block (cAVB) was detected in 2 (2.1%) patients with perimembranous VSD closed with ADO I. A transient block was detected on the first day after the catheterization in one patient and on the third day in the other; both resolved on prednisolone by the fifth- and seventh days post-procedure without temporary pacing. None of our cases had serious events and no short-term mortality.

**Fig. 4:**
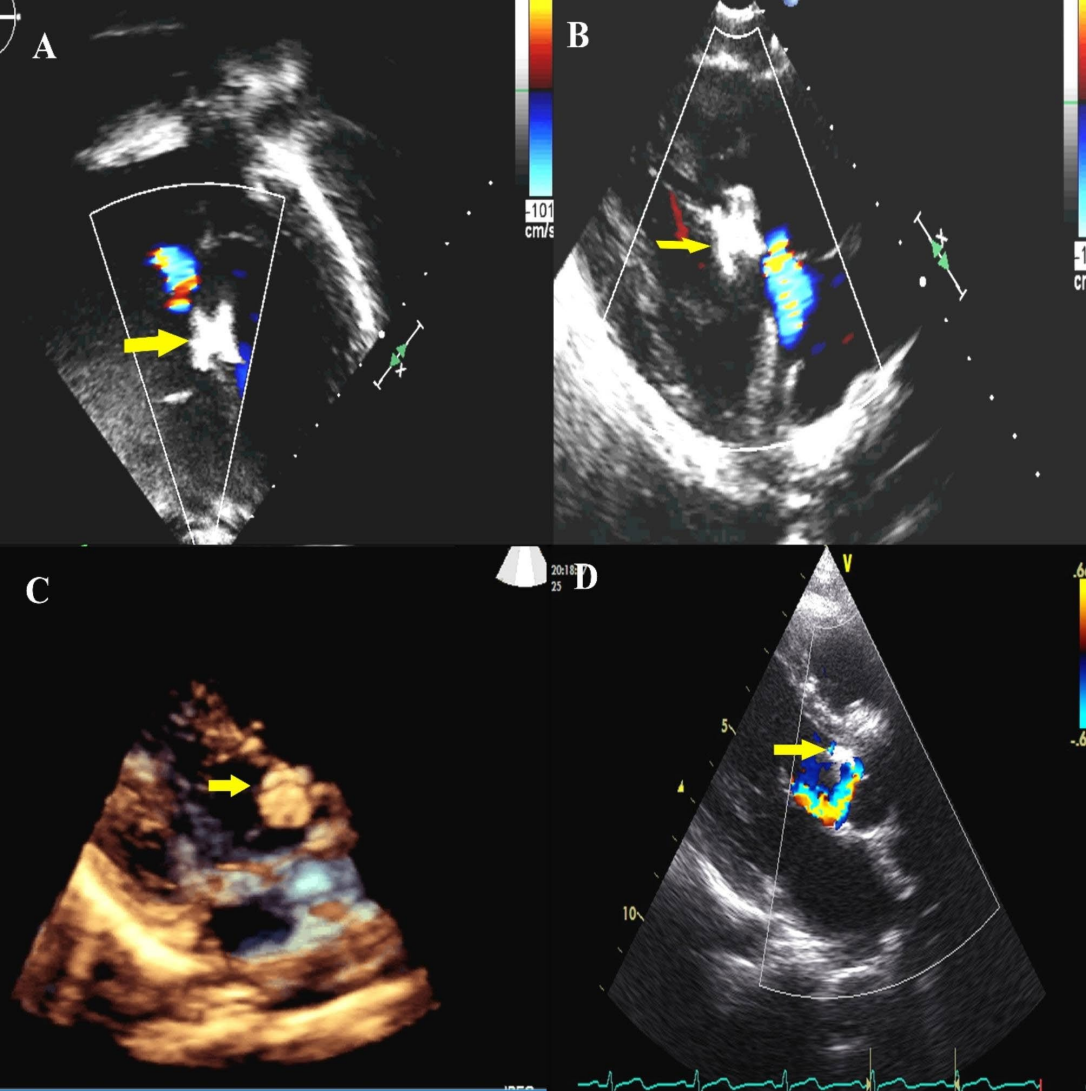
Long-term follow-up using transthoracic echocardiography: **(A, B)** Two-dimensional four-chamber view and left parasternal long-axis view (in rightward and inferior angulation toward right hip) demonstrating mild tricuspid regurgitation caused by Hyperion muscular device (yellow Arrow) used to close a high muscular outlet VSD **(C)**, Three-dimensional parasternal long-axis view of ADO I is seen slightly protruding in the LVOT causing minimal aortic regurgitation and **(D)** Long parasternal view demonstrating aortic regurgitation caused by a device in closing the perimembranous VSD.

On long-term follow-up, two cases had persistence of the new tricuspid regurgitation, three resolved on follow-up, and three patients had persisted residual shunt. However, none of them showed hemodynamic consequences. One case with mild aortic regurgitation early post-procedure progressed to severe regurgitation by the end of the second year, causing heart failure in the early third year of follow-up. Surgical removal of the device and patch closure of VSD was performed. The surgeon reported that the valve’s right coronary cusp was trapped underneath the device. However, the valve did not require any intervention, as the regurgitation was less upon device removal. No new emerging complication was documented on long-term follow-up and no mortalities. Echocardiographic images of some long-term encountered complications are demonstrated in Fig. 4.

Table 4 compares different devices used for VSD closures regarding demographic, device size, complications, and long-term follow-up. Although no statistically significant results were found regarding the age, sex, and weight of patients at the time of device implantation, significant differences were detected in the size of the device and follow-up duration (P-value = 0.0001). The largest device sizes were used for Muscular Amplatzer, followed by Hyperion. The longest follow-up duration was for ductal occluders and Amplatzer muscular devices, and the least was for Occlutech. Primarily it could be attributed to Occlutech being a recently released device used in the most recent patients. The immediate residual flow was the highest following coil in 28% of cases; however, this rate declined to 3.1% on follow–up. The highest incidence of new tricuspid regurgitation was detected with Hyperion devices in 9.5% of cases, while the ADOI was the most frequently implicated in new post-procedural aortic regurgitation 2 (18.2%).

**Table 4:** Comparison between devices regarding demographic, device size, complication, and long-term follow-up

	Pfm Coil^#^ N = 32	Hyperion^#^ N = 21	ADO I^#^ N = 11	ADO II^#^ N = 1	AMO^#^ N = 8	Occlutech^#^ N = 2	P-Value
Age	10(8.63-12)	9 (6.5–12)	9 (5–10)	7	8.5 (6.25–10.75)	4, 8.5	0.344
Sex (M)	19 (59.4)	10 (47.6)	5 (45.5)	1 (100)	5 (62.5)	1 (50)	0.83
Weight	36 (23.3–45.8)	30 (19.5–38)	21 (17–33)	21**	25 (19.5–49)	12, 22.5**	0.16
Device Size ^##^	6 (6–8)	10 (10–12)	6 (4–6)	4**	14 (14–18)	6, 8**	0.0001*
Follow-Up Duration	67 (66–96)	45 (42–46)	110 (96–112)	112**	96 (62-114.2)	36, 49**	0.0001*
Immediate Residual	9 (28)	1 (4.8)	2 (18.2)	1 (100)	1 (12.5)	-	-
Long-term residual	1 (3.1)	1 (18.2)	0 (0)	1 (100)	-	-	-
New TR	1 (3.1)	2 (9.5)	1 (9.1)	-	-	1 (50)	-
New AR	-	1 (4.8)	2 (18.2)	-	-	-	-

**ADO**: Amplatzer duct occluder, **AMO**: Amplatzer Muscular VSD Occluder, **AR**: aortic regurgitation, **M**: male, **TR**: tricuspid regurgitation, **VSD**; ventricular septal defect

^#^ Data are expressed as median (interquartile range) or number (percentage)

^##^ Device size equals waist diameter in mm except for coil and ADO I; it was the diameter of the device’s right end in mm

* P-value significant if ≤ 0.05

** Not included in statistical comparison because of limited patients (less than 5 patients)

## Discussion

The percutaneous closure of VSDs has become a standard practice utilizing different occluding devices, enabling an expansion in managing various types and morphologies of VSD. The current work thoroughly investigated the outcome of transcatheter closure of VSD using six types of devices over a long duration of follow-up, reaching up to 9.5 years in some cases.

### Residual shunt

The technical success in our series was achieved in 97.9% of patients, the immediate success rate was 84%, and the maximum achieved success rate was 95.7% at one year without further complete closure beyond a year of follow-up. The highest rate for closure was 100% for AMO, and the lowest rate for immediate closure was for the Pfm Nit-occlud LeˆVSD coil, which improved on the follow-up to a closure rate consistent with other devices. In agreement with our results, a success rate of 91% was detected in the Rahmath et al. cohort after 54.5 months of follow-up [17]. A lower ultimate closure rate than ours was reported by Bergmann et al. (86.2%), with the highest rates on using Amplatzer membranous and muscular occluders (93–95%) and the lowest with Nit-Occlud LeˆVSD coil at (61%); with a new small shunt detected at 40 weeks of gestation in a pregnant patient three years after using the coil [20]. In EUREVECO Registry, Nit-occlud LeˆVSD coil’s immediate closure rate was reported to be as low as 50%, but increased to 97% after one year [14]. Nevertheless, Walavalkar et al. found that the probability of device failure was not associated with device type, as they did not detect a significant difference in failure rate between muscular and ductal devices [21]. For residual post-operative VSDs, we had successful closure in 10/11 (90.9%) patients; only one had a small residual. Comparable results were reported by Taha et al. with successful device closure of 18 residual VSDs either post-operative or post-catheterization with no reported complication or mortality after a mean follow-up of 23.3 months [22].

### Tricuspid regurgitation

New-onset trivial/mild tricuspid regurgitation was diagnosed early after device implantation in 5.3% of our cohort without progression, with a resolution of the regurgitation in two patients. In contrast, Rahmath et al. documented a higher rate of new tricuspid regurgitation immediately post-procedural in 18 (40%) with a resolution of mild regurgitation in seven patients, while moderate regurgitation persisted at 54.5 months of follow-up [17]. On the contrary, a lower rate of mild degree tricuspid regurgitation was detected by Mandal et al. in 1.1% of their patients [10]. However, progression from mild degree post-procedure to severe tricuspid regurgitation causing heart failure was reported three years following transcatheter VSD closure due to coaptation failure of the valve leaflets as a result of septal leaflet entrapment by the device [23]. Similarly, severe tricuspid regurgitation requiring surgical repair with coil removal was reported in two other cases of perimembranous VSD closed with Nit-Occlud LeˆVSD Coil [19]. Impingement of the device on the septal leaflet of the tricuspid valve and rupture of the chordae tendineae are possible etiologies of regurgitation in rare situations. Therefore, the abnormal origin of the tricuspid valve main chordae tendineae from a perimembranous VSD was screened for and was considered an exclusion criterion for transcatheter closure in some centers [24].

### Aortic regurgitation

Another VSD occluders-related complication is aortic valve regurgitation. Our study documented new-onset regurgitation in 3 (3.2%) patients early post-procedure. Only one case progressed to a severe degree, requiring surgery. In contrast, Han et al. reported aortic regurgitation in 0.1% of their cohort; four patients had severe regurgitation 9–12 years post-VSD transcatheter closure requiring replacement [25]. Another report documented aortic regurgitation after four years with no hemodynamic consequence in 1 (0.6%) case [10]. On the contrary, a high incidence of new immediate trivial/mild aortic regurgitation was reported in 5/45 (11.1%) patients in another study, increased to 13.3% on long-term follow-up for 54.5 months; however, none exceeded a mild degree of regurgitation [17]. Similarly, Walavalkar documented new immediate aortic regurgitation in 15% of patients, reduced to 5% at a median follow-up of 246 days using 15 different devices [21]. New-onset or worsening aortic regurgitation was the primary cause of unplanned surgery after VSD transcatheter closure in children [26]. Aortic regurgitation could result from iatrogenic cusp injury or perforation during the placement or retrieval of a device [27]. Furthermore, it could be the occluder effect on the aortic valve as a large occluder may get too close to the aortic valve, and the memory alloy of an occluder may gradually erode the surface in contact, causing delayed aortic regurgitation [28, 29].

### Arrhythmia

In the current work, the rate of early post-procedure arrhythmia was 4.3%; only two patients with perimembranous VSD closed using ADO I had early cAVB resolved on steroid therapy without pacing. However, no atrioventricular block (AVB) was detected on prolonged tracking of cases. Unlike our data, Zhao et al. noted high arrhythmia rates, reaching 24.1% early following transcatheter closure; 77.8% reverted to sinus rhythm during 35.5 months of follow-up. On logistic regression, they found a significant relation between arrhythmia and long fluoroscopy time when using eccentric or large devices [30]. Another study on perimembranous VSD found that 25.5% of cases had early arrhythmia post-device insertion with 2.7% serious arrhythmias, including second & third AVB and left bundle branch block (LBBB), with reported late onset in seven patients (6 months to 8.3 years later) [31]. Another series reported that 8.5% of patients had conduction abnormalities; with transient cAVB occurring in two patients, junctional rhythm (27 patients), RBBB (3 patients), and LBBB (2 patients) [31]. Bergmann et al. 4/109 (3.7%) found variable arrhythmias after one year of follow-up, including one case of SVT [20].

The incidence of cAVB associated with transcatheter closure of perimembranous VSD was reported to be 1–6%, depending on different clinical experiences and occluder selection [32]. A meta-analysis of percutaneous device closure of pmVSD revealed that the incidence was 1.1% [33]. The cAVB rate was 0.7%, as reported by Bergmann et al., with no other reported arrhythmia within six years of follow-up for six different devices [20]. However, Li et al. reported a higher rate of early conduction abnormalities after perimembranous VSD device 19/79 (24.05%); 11 cases of incomplete RBBB (6 resolved on 35.3 ± 17.4 months follow-up) and 5 cases of complete RBBB (one resolved on long-term follow-up), and reversible cAVB; two of them received temporary pacemaker implantation. These patients recovered one, six, and nine days later with no new reported cases on long-term follow-up for 3–5 years [34]. Nevertheless, a new AVB could develop years after device insertion; as Xie et al. reported, a cAVB case was diagnosed 2.5 years post perimembranous VSD closure using a modified double-disc VSD occlude [35]. Also, Bai et al. reported that three patients developed AVB beyond one year of VSD device, one of these developed 5.3 years post-procedure [36]. It was suggested that AVB occurring immediately after the procedure might result from direct mechanical compression by the device or inflammatory edema of the membranous interventricular septum near the AV node and conduction branches. However, late block after weeks or months post-procedural may result from a localized inflammatory reaction caused by the device that can result in extensive scar tissue and cartilaginous metaplasia of the surrounding myocardium.). Another proposed mechanism for late cases of AVB is device flattening [37, 38]. Therefore, the early block tends to respond to steroid therapy, while the late block requires management with a permanent pacemaker. A single report described a rare recurrent cAVB 42 months after the VSD transcatheter closure complicated with transient cAVB one week after the procedure [39].

The proximity of the membranous septum to the septal leaflet of the tricuspid valve on the VSD right ventricular side and the aortic valve on the left ventricular side, with its inferoposterior margin closely related to the bundle of His and bundle branches, is considered a challenging anatomy. Therefore, successful closure of the VSD transcatheter requires complete anatomic delineation of the defect, careful VSD sizing, and careful determination of the relation to surrounding cardiac structures for proper device selection to minimize the risk of complications. On the one hand, undersized devices are associated with device embolization and residual shunt. On the other hand, oversized devices may damage adjacent structures, causing cAVB or injury of aortic or tricuspid valves [40].

### Infective endocarditis

No cases of infective endocarditis were detected in the current series through the follow-up duration. However, rare cases were reported in the literature. In one case, early VSD device-related infective endocarditis was reported in a patient ten days after a Nit-Occlud LeˆVSD coil insertion [41]. A case of pulmonary valve endocarditis was reported by Carminati et al. a few days post-implantation of two Amplatzer VSD muscular occluders [42]. Another case was diagnosed with Kingella kingae endocarditis four months after transcatheter closure of perimembranous VSD using a Nit-Occlud Leˆ VSD coil [43]. Late-onset infective endocarditis could occur following device closure if associated with post-procedure aortic regurgitation, as reported by Tang et al. 11 years post-VSD closure using symmetric double-disk occluder (SHAMA) [44].

### Mortality

No device-related mortality for percutaneous closure of VSD was documented in the current series post-procedure or on the long-term follow-up that was extended up to 9.5 years. Our finding is consistent with several published studies reporting no deaths on short- or long-term follow-ups for percutaneously implanted devices [14, 20, 45, 46]. Limited cases of early procedure-related mortalities were documented, such as Carminati et al. report of one intraprocedural mortality in a case during second AMO implantation, resulting in a mortality rate of 0.2% [42]. Moreover,  there is a report of one patient died five days following catheterization due to intracranial bleeding [21]. Furthermore, Jiang et al. reported two (0.3%) deaths; one developed subarachnoid hemorrhage due to cerebral vascular malformation, whereas the other was arrested secondary to cAVB 40 days post-procedure. However, they did not document additional mortalities on a long-term follow-up duration of 46 months [47].

Although the current study proves the favorable outcome of VSD percutaneous closure after several years of follow-up, the study design has inherent limitations. One of the primary limitations was the retrospective design of the study. Furthermore, the results might not represent those encountered in different centers as it is a single-center experience. Moreover, the patients’ sample size was insufficient for some devices for a solid statistical analysis. Therefore, future prospectively designed studies with larger sample sizes and the involvement of multiple centers are fundamental to confirm the long-term outcome of variable devices used in VSD percutaneous closure.

## Conclusion

In conclusion, according to our experience, transcatheter closure of VSD is safe in pediatric patients with no related mortality in immediate or long-term surveillance. Long-term safety is comparable among different devices. Although post-procedural adverse events were limited, extended follow-up is required for cases with early reported complications for the limited potential of progression to significant morbidity, especially the aortic valve regurgitation.

## Data Availability

The data are not publicly available because they contain information that could compromise the privacy of research participants in this study. Data is available from the corresponding author upon reasonable request.
